# Timing of surgery and elective perioperative management of patients with previous SARS-CoV-2 infection: a SIAARTI expert consensus statement

**DOI:** 10.1186/s44158-022-00058-3

**Published:** 2022-06-22

**Authors:** Andrea Cortegiani, Vincenzo Francesco Tripodi, Carlo Alberto Castioni, Clelia Esposito, Nicola Galdieri, Roberta Monzani, Marco Rispoli, Alessandro Simonini, Vito Torrano, Antonino Giarratano, Angelo Gratarola

**Affiliations:** 1 Department of Surgical, Oncological and Oral Science (Di.Chir.On.S.), University of Palermo, Palermo, Italy; 2 Department of Anesthesia, Intensive Care and Emergency, Policlinico Paolo Giaccone, University of Palermo, 90127 Palermo, Italy; 3 Department of Surgery, Anesthesia and Intensive Care Unit, Policlinico Gaetano Martino, University of Messina, Messina, Italy; 4 IRCCS, Istituto Delle Scienze Neurologiche, UOC Anestesia E Rianimazione, Bologna, Italia; 5 Critical Area Department, Anesthesia and Intensive Care, AO Dei Colli, Vincenzo Monaldi Hospital, Naples, Italy; 6 Critical Area Department, Intensive Care in Cardiac Surgery, Vincenzo Monaldi Hospital, Naples, Italy; 7 Department of Anesthesia and Intensive Care, IRCCS Humanitas Research Hospital, Rozzano, Milan Italy; 8 Pediatric Anesthesia and Intensive Care Unit, Salesi Children’s Hospital, Ancona, Italy; 9 Department of Emergency and Urgency, Anesthesia and Intensive Care 1, ASST Grande Ospedale Metropolitano Niguarda, Milan, Italy; 10 Department of Emergency, Anesthesia and Intensive Care Unit, IRCCS for Oncology and Neuroscience, San Martino Policlinico Hospital, Genoa, Italy

**Keywords:** SARS-CoV-2 infection, COVID-19, Elective surgery, Vaccination, Long COVID, Complications, Perioperative morbidity and mortality

## Abstract

**Background:**

The appropriate timing of surgery and perioperative management of patients with previous SARS-CoV-2 infection are open issues. The purpose of this document is to support the clinical decision-making process regarding the patient with previous Sars-CoV-2 infection to undergo elective surgery. The recipients of this document are physicians, nurses, healthcare personnel, and other professionals involved in the patient’s surgical process.

**Methods:**

The Italian Society of Anesthesia Analgesia Resuscitation and Intensive Care (SIAARTI) selected 11 experts to reach a consensus on key aspects of this theme in adult and pediatric population. The methods of this process document were in accordance to the principles of rapid review of the scientific literature and modified Delphi method. The experts produced statements and supporting reasons in the form of an informative text. The overall list of statements was subjected to a vote in order to express the degree of consent.

**Results:**

Patients should not undergo elective surgery within 7 weeks of infection unless there is the risk of a negative evolution of the disease. To mitigate the risk of postsurgical mortality, a multidisciplinary approach seemed useful in addition to the use of validated algorithms to estimate the risk of perioperative morbidity and mortality; the risk related to SARS-CoV-2 infection should be added. The risk of potential nosocomial contagion from a positive patients should also be considered when deciding to proceed with surgery. Most of the evidence came from previous SARS-CoV-2 variants, so the evidence should be considered indirect.

**Conclusion:**

A balanced preoperative multidisciplinary risk–benefit evaluation is needed in patients with previous infection by SARS-CoV-2 for elective surgery.

**Supplementary Information:**

The online version contains supplementary material available at 10.1186/s44158-022-00058-3.

## Background

The perioperative management of patients who have contracted previous infection with SARS-CoV-2, scheduled for elective surgery, is an important clinical-organizational problem. The infection constitutes a potential additional risk of the onset of potentially severe perioperative complications.

The Italian Society of Anesthesia Analgesia Resuscitation and Intensive Care (SIAARTI) therefore decided to create a panel of experts aiming to draft consensus statement that outlines clinical suggestions on this matter, in light of the most recent evidence, available guidelines [1], and current uncertainties in both adult and pediatric population. At the moment, the statements suggest not to treat patients with elective surgery within 7 weeks of infection unless there is a substantial risk of disease progression. In order to mitigate the risk of postsurgical morbidity and mortality, a multidisciplinary approach is useful in addition to the use of validated calculation algorithms, to estimate the basal risk. The new variants, Omicron and sub-variants, are characterized by a very high contagion rate. Elective surgical interventions in subjects who have developed severe forms of infection or who still have related symptoms could be extended beyond the standard 7 weeks. However, it is reasonable not to undergo surgery within 10 days of positivity to SARS-CoV-2 tests for aspects related to infectivity, the difficulty in the pathway of care, and not least to the potential evolution of the infection. It is also reasonable to assume that a 10-day delay for an elective surgical procedure does not imply a worsening of the expected outcomes.

Therefore, final decision on perioperative management should be weighted considering the combination between the measurement of baseline risk, the specific clinical conditions, and the vaccination status of the subject that should be completed preoperatively, representing a valid tool of clinical support for patient protection.

## Methods

The experts, members of the panel, were selected by one coordinator (Angelo Gratarola), according to specific skills and clinical experience in order to reach consensus on key aspects of the perioperative path of patients with previous SARS-CoV-2 infection. The project was promoted by independent requests from numerous members and by the presidency of SIAARTI. The overall consensus process was coordinated by a clinician with expertise in methodology (Andrea Cortegiani).

### Methodology

During the first kick-off meeting, the standard methodology adopted by SIAARTI for consensus process was shared, and main themes were openly discussed. Then, the main selected topics were assigned to one or more panel members, on the basis of their respective skills, in order as follows:

1. Evaluate the available evidence
2. Produce statements and supporting rationales in the form of explanatory text.

The overall list of statements was subjected to a blind vote, according to the established scientific method, in order to test consensus.

The methodological path of the document was outlined by a methodologist (Andrea Cortegiani) and was based on the principles of *rapid review* of the scientific evidence and of the modified Delphi method for evaluating consensus. Specifically, the search strategy was created by two experts on the subject (Andrea Cortegiani, Vincenzo Francesco Tripodi), with defined time limits (1 October 2021–10 March 2022), on PubMed, using MeSH words (“COVID” and “surgery”). Full search strategy [E-Table 1] and the inclusion/exclusion process according to PRISMA 2020 flow chart [Fig. 1] can be found in the Additional file 1.

The choice of the time restriction was based on the following:

1. The rapid review principles adopted in light of the urgent nature of the issue [2]
2. On the fact that an appropriate guideline was already available, taken as a reference by the panel for methodological quality and contents, which had already evaluated the previously available literature [1]

Eligible article was as follows: original articles, case series, case reports, narrative reviews, systematic reviews, meta-analysis, position papers, and guidelines. Non-English articles and conference proceedings were excluded. The search and its reporting were conducted according to the principles of PRISMA 2020 [3]. The method considered a maximum of two possible rounds of online blind voting. The panelists casted their vote blindly in the votes made. The opinion was expressed using an ordinal Likert scale, according to the ULAS-RAND method (minimum score, 1 = completely disagree; maximum score, 9 = completely agree) [4]. This scale was divided into 3 sections: 1–3 implied refusal/disagreement (“inappropriate”), 4–6 implied “uncertainty,” and 7–9 implied sharing/support (“appropriateness”). Consensus was reached when as follows:

1. At least 75% of the respondents (excluding the methodologist and the search specialist) assigned a score in scores 1–3, 4–6, or 7–9, which meant refusal or sharing of the statement, respectively.
2. The median of the score was within the same range. The type of consensus was determined by the positioning of the median. It was not necessary to run the second Delphi round, as all statements reached consensus at the first round. The results of the votes were reported in tabulated form. The full version of the Italian document issued by SIAARTI was published in March 2022 [5].

## Results

### Preliminary remarks — adult patient

Preoperative SARS-Cov-2 infection was previously known to be associated with a significant increased risk of morbidity and mortality. Data from the early phase of the pandemic showed that a perioperative infection from SARS-Cov-2 was clinically associated with an important increase in mortality, in some cases, as much as 10 times [6, 7]. Furthermore, when surgery was undertaken within 6 weeks of infection, both postoperative morbidity and mortality increased [8]. In particular, the increase in perioperative risk remained consistently high for up to 7 weeks after the SARS-CoV-2 infection. Only after that period of time, it returned to baseline. Therefore, indications have been made to postpone elective surgery after SARS-CoV-2 infection by at least 7 weeks unless the risks of postponing surgery outweigh the risk of associated postoperative morbidity or mortality to infection with SARS-CoV-2 [1, 9]. As the COVID-19 pandemic progresses, both drug and vaccine preventive therapies have been developed [5]. Variants have emerged that differ both in transmissibility and in the severity of the disease they cause and in their ability to infect vaccinated patients. In particular, the Omicron SARS-CoV-2 has increased transmissibility and the ability to evade immunity acquired with previous SARS-CoV-2 infections and with vaccination or through both options [10]. This variant also results in a clinically less severe disease than the previous variants [11, 12].

Compounding this situation, the reduction in surgical care delivery during the pandemic has significantly increased the number of patients awaiting surgery globally [13, 14].

In light of these uncertainties and of the expectation of the imminent return of a surgical activity with increased volumes, the clinical decision-making process regarding the “timing” of the surgery has become decisive [15].

The goal of this paper is to provide an update with respect to previous documents on SARS-CoV-2 infection, COVID-19, and the timing of elective surgery; in order to support the legislator, political institutions, healthcare personnel, and patients, the underlying principles of this paper may also be relevant for future variants.

### Preliminary remarks — pediatric patient

During the SARS-CoV-2 pandemic, the provision of the pediatric surgery service was heavily reduced. Most centers canceled or postponed nonurgent surgeries while maintaining neonatal surgery [10]. A significant impact has been observed on surgical approaches to the pediatric patient with a significant reduction in all procedures deemed capable of generating aerosols [16]. SARS-CoV-2 binds to the receptors of the angiotensin-converting enzyme II (ACE II) to access the host organism, and the expression of these receptors depends on age, being relatively lower in children than in adults. Consequently, children over 1 year of age are characterized by a lower risk of severe or moderate infection. This is also due to an immune response not yet fully developed in the child and, therefore, to a reduced possibility of the immune-mediated damage typical of COVID-19 [17]. Infants younger than 1 year of age may have a higher risk of developing severe or moderate infection because their defenses depend on maternal antibodies [18].

Mortality due to COVID-19 in the COVID-19-positive pediatric population was estimated to be 0.005% [14, 19, 20]. To date, children with perioperative SARS-CoV-2 infection do not appear to be at increased risk of postoperative lung complications or mortality; however, there is insufficient evidence to draw absolute conclusions [21–24].

### Rationale of the perioperative pathways for SARS-CoV-2

The planning of the perioperative pathways in the SARS-CoV-2 era must consider epidemiological changes of the infection over time. Despite the lower perioperative mortality described for the Omicron variant, it remains mandatory to keep the incidence of hospital infections at the lowest possible level. To achieve this goal, careful planning is needed, especially due to the high contagion rate associated with the Omicron variant.

The planning of adequate pathways allows on the one hand to reduce the rate of infections and on the other guarantees the use of health resources for the restoration of elective surgical activity. The reduction of surgical activities could represent a significant pitfall for public health expenditure, entailing a potentially greater cost than managing the patient with SARS-CoV-2 [25].

### Perioperative vaccination — adult patient

Access to vaccination represents the main form of prevention of SARS-CoV-2 infections and, consequently, of postoperative complications related to the infection. Particular attention should be paid to clinically vulnerable patients such as candidates for cancer surgery and patients over the age of 70 [26]. Indeed, it is widely demonstrated that COVID-19 mortality is significantly reduced in vaccinated patients undergoing surgery, compared to the general vaccinated population [27]. The third dose of vaccine, on the other hand, significantly reduces both the risk of infection and the severity of the disease [11, 28]. Full clinical efficacy of the vaccine usually occurs within 2 weeks of administration. Therefore, providing, as part of the preoperative pathway, a third administration to the still only partially vaccinated patient can have a significant impact on the postoperative period [29]. However, some typical vaccination side effects should be considered for their potential impact on surgical outcome, for which several authors recommend wait at least 3 weeks after vaccination before undergoing elective surgery. This measure serves both to avoid any diagnostic confusion about the cause of symptoms such as fever (a consequence of vaccination or complication of the surgery?) and to allow sufficient time for the formation of antibodies [30, 31].

### Perioperative vaccination — pediatric patient

The recent availability of vaccines for the pediatric population aged 5 years and over could reduce the already low incidence of severe forms in children and, consequently, the perioperative risk of patients with a history of previous infection. It is important to minimize the chances of contagion in the weeks preceding the scheduled surgery with vaccination (children over 5 years). Considering that the pediatric patient could be poorly cooperative, prevention measures should be provided to parents in the preoperative indications through adequate information material to be delivered during the pre-hospitalization assessments.

### Focus on the patient

It is essential to communicate to the patient, to his family, and to those who care for here/him, in a complete and exhaustive way, the reasons for which the intervention is eventually postponed, thus reducing the sense of medical abandonment [32]. Keeping in touch with patients waiting is also useful in order to encourage the following:

- Use of the mask
- Social distance
- Hand hygiene
- Preoperative self-isolation as appropriate [33]

Patients on the waiting list for surgery often report the onset of anxiety that could worsen if not treated, especially in the case of time-dependent procedures. In these settings, anxiety and depression are common and are related to higher mortality than less time-dependent procedures (for example, cataracts or joint replacement), despite having a significant impact on quality of life [34, 35]. Finally, periodic communication on the position on the waiting list, the priority rules, and the expected date for the procedure can improve the patients’ experience, their background, and, in some cases, the clinical outcomes [36]. In this regard, telemedicine can be one of the resources for maintaining the engagement of patients on the waiting list. The ASA (American Society of Anesthesiologists) reports that many anesthesiologists have used telemedicine during the COVID-19 pandemic [37]. While precluding direct physical evaluation of the patient, telemedicine allows information to be collected even before patient admission, such as the possible presence of other comorbidities, the potential evaluation of the surgical intervention as urgent, and also the selection of the appropriate anesthetic and analgesic technique [26, 38, 39].

In addition to relieving patient anxiety, telemedicine is also useful in minimizing the time spent by patients in healthcare settings. Among the other perioperative organizational measures, considering the reduction of resources both in the wards and in the intensive care units, the implementation of enhanced recovery after surgery (ERAS) pathways appears appropriate [40]. An ERAS program has the potential objective to improve the outcome after surgery; to promote a timely postoperative discharge, shortening the time of stay of patients in hospital; to reduce complications; and, therefore, to increase the potential resources that can be allocated, in case of need, to the treatment of COVID-19 patients [40, 41].

### Focus on healthcare staff

Mass screening for healthcare workers can reduce both unnecessary quarantine and the “silent” spread of mild or asymptomatic cases, avoiding workforce depletion and reducing intrahospital transmission [33]. Screening of hospital staff is therefore essential, as is the use of personal protective equipment (PPE) and the adoption of appropriate programming criteria, which also make use of the aid of telemedicine [42].

### Focus on the hospital organization

It is necessary to ensure adequate ventilation of hospital environments and adequate air filtration. Loganathan et al. [43], in their depth review, recommend among other things the following:

- Ensure negative pressure in the operating room
- Ensure frequent air circulation
- Allow access to the operating room to the necessary health personnel only

### Focus on the postoperative

It is also essential to maintain dedicated perioperative pathways, with patients separated based on the results of the screening. If postoperative patients develop symptoms suggestive of COVID-19, a polymerase chain reaction test should be performed. In particular, Prasad et al. report that female patients with end-stage renal disease, congestive heart failure, COPD, liver cirrhosis, cancer, or undergoing neurosurgical procedures are more likely to test positive for SARS CoV-2 after elective surgery [44, 45]. It could also be useful to manage the periodic remote follow-up.

### Statements for adult patient

1. There is now no evidence regarding perioperative outcomes after SARS-CoV-2 vaccination and the Omicron variant. Patients should not undergo elective surgery within 7 weeks of SARS-CoV-2 infection, unless the benefits of performing the surgery are greater than the expected risk. A personalized and individualized risk assessment is recommended for patients who must undergo elective surgery within 7 weeks of SARS-CoV-2 infection.
  [Median score: 9 (*IQR* 7–9); agreement: 8/9 (88.8%)].
2. Preoperative vaccination for COVID-19 is recommended, possibly with three doses. The last dose should be administered at least 2 weeks before surgery.
  The certification and completion of the vaccination plan should be carried out as soon as possible, both for the start of primary care and for the planning of the surgery itself.
  [Median score: 9 (*IQR*: 7–9); agreement: 9/9 (100%)].
3. Current measures to reduce patients risk for SARS-CoV-2 infection in the perioperative period should continue and, given the increased transmissibility of Omicron variant, where evidence supports it, should be implemented (e.g., the use of individual protective devices for the respiratory tract).
  [Median score: 9 (*IQR*: 7–9); agreement: 9/9 (100%)].
4. Patients should notify the surgical team they are positive for SARS-CoV-2 infection test result within 7 weeks from the planned date for surgery. In this case, it is recommended to organize a meeting between the perioperative team and the patient to discuss risks and benefits of postponing surgery.
  [Median score: 9 (*IQR*: 7–9); agreement: 9/9 (100%)].
5. A patient positive to SARS-CoV-2 test should not undergo elective surgery within 10 days from diagnosis. Patient may be infectious and could represent a risk for surgery, team, and other patients.
  [Median score: 9 (*IQR* 7–9); agreement: 8/9 (88.8%)].
6. There is now no evidence on the perioperative risk due to infection caused by Omicron variant. The hypotheses that the infection, asymptomatic or mildly symptomatic, does not add risks are now unfounded.
  [Median score: 9 (*IQR*: 7–9); agreement: 9/9 (100%)].
7. We recommend a multidisciplinary discussion with the patient, documenting the risks and benefits of surgery, in case is not possible to refer the elective surgery over 7 weeks of the diagnosis of SARS-CoV-2 infection.
  - All patients should know their risk of mortality and complications using a validated risk score.
    [Median score: 9 (*IQR*: 7–9); agreement: 9/9 (100%)].
  - To estimate the perioperative risk of surgery within 7 weeks of infection, it is reasonable to consider the following “risk modifiers” and risk factors: patient factors such as age and comorbidities; SARS-CoV-2 infection such as timing, severity of initial infection, and the presence of symptoms; clinical priority; risk of disease progression; and complexity of surgery.
    [Median score: 9 (*IQR*: 7–9); agreement: 9/9 (100%)].
  - It is recommended to inform the patient that the decision to proceed with surgery within 7 weeks will be not evidence-based.
    [Median score: 9 (*IQR* 7–9); agreement: 8/9 (88.8%)].
8. Patients with moderate to severe infection and with persistent symptoms are likely to be at increased risk for morbidity and mortality, even after 7 weeks. Therefore, it is recommended to postpone surgery beyond this period, balancing this risk with any other associated with this delay.
  [Median score: 8 (*IQR*: 7–9); agreement: 9/9 (100%)].
9. In patients with recent or perioperative SARS-CoV-2 infection, it is reasonable to consider avoiding general anesthesia in favor, where possible, of local or regional anesthesia techniques.
  [Median score: 9 (*IQR* 7–9); agreement: 8/9 (88.8%)].
10. Rather than considering only the time interval between infection and surgery, it is important to assess the timing, the patient’s basic assessment, and the increased risk and to share the multidisciplinary decision-making.
  [Median score: 9 (*IQR* 7–9); agreement: 8/9 (88.8%)].
11. All patients awaiting surgery should be encouraged to undergo good practice of preoperative optimizations: nutritional optimization, stopping active smoking, and physical training. They should be also encouraged to comply with preventive measures that reduce the risk of community-acquired SARS-CoV-2 infection.
  [Median score: 8 (*IQR*: 7–9); agreement: 9/9 (100%)].

### Statements for pediatric patient

1. There is currently no evidence regarding perioperative outcomes after vaccination for SARS-CoV-2 and after the appearance of the Omicron variant.
  If possible, patients should not undergo elective surgery during the 7 weeks following SARS-CoV-2 infection, unless the benefits outweigh the expected risk. A personalized risk assessment is recommended for pediatric patients who need to undergo elective surgery within 7 weeks of SARS-CoV-2 infection.
  [Median score: 9 (*IQR*: 7–9); agreement: 9/9 (100%)].
2. Surgical pediatric patients over 5 years of age should have preoperatively received vaccination, possibly with two doses. The last administration of the vaccine should occur at least 2 weeks before surgery. Certification and completion of the vaccination plan should be done as soon as possible, both for referral to primary care and for surgery.
  [Median score: 9 (*IQR*: 7–9); agreement: 9/9 (100%)].
3. Current measures reduce the risk of hospital-acquired infections in the perioperative period should continue, and, given the increased transmissibility of the Omicron variant, where the evidence supports it and there is the possibility, personal protective measures should be implemented (e.g., with the use of respiratory protective devices).
  [Median score: 9 (*IQR* 7–9); agreement: 8/9 (88.8%)].
4. If the child tests positive for SARS-CoV-2, parents or legal guardians should notify the multidisciplinary team, within 7 weeks of the scheduled surgery date. If the child tests positive for SARS-CoV-2, it is recommended to organize a meeting between the multidisciplinary team and the patient’s parents/legal guardians in order to share the risks and benefits of postponing the surgery.
  [Median score: 9 (*IQR*: 7–9); agreement: 9/9 (100%)].
5. Elective surgery should not occur within 10 days of being diagnosed with SARS-CoV-2 infection, primarily because the infected patient poses a risk to the surgery, the medical staff, and other patients.
  [Median score: 9 (*IQR* 7–9); agreement: 8/9 (88.8%)].
6. There is no evidence on the impact that asymptomatic or mildly symptomatic infection could have on perioperative risk. Although the mortality of infected pediatric patients appears extremely low, the hypothesis that asymptomatic or mildly symptomatic infection does not add risk is currently unfounded.
  [Median score: 9 (*IQR*: 7–9); agreement: 9/9 (100%)].
7. When considering elective surgery within 7 weeks of diagnosis of SARS-CoV-2 infection, it is recommended to have a multidisciplinary discussion with the patient’s parents/legal guardians, documenting the risks and benefits, including the following:
  - The risk of mortality (and complications where possible) should be calculated using a validated risk score for all patients.
    [Median score: 9 (*IQR*: 7–9); agreement: 9/9 (100%)].
  - Risk modifiers based on patient factors (age, comorbid status); SARS-CoV-2 infection (timing, severity of initial infection, current symptoms); and surgical factors (clinical priority, risk of disease progression, complexity of surgery) can, therefore, be applied to help estimate how the underlying risk might vary by undertaking surgery within 7 weeks of infection.
    [Median score: 9 (*IQR*: 7–9); agreement: 9/9 (100%)].
  - The patient’s parents/legal guardians should be advised that the decision to proceed with the surgery within 7 weeks will be pragmatic, rather than based on scientific evidence.
    [Median score: 9 (*IQR* 7–9); agreement: 8/9 (88.8%)].
8. Patients with persistent symptoms and patients with moderate or severe COVID-19 (e.g., those who have been hospitalized) are likely to be at increased risk for morbidity and mortality, even after 7 weeks. Therefore, surgery should be postponed beyond this period, balancing this risk with any other associated with this delay.
  [Median score: 9 (*IQR*: 7–9); agreement: 9/9 (100%)].
9. General anesthesia, AG or local anesthesia, AL or locoregional anesthesia, and ALR with associated sedation should be performed in patients with recent or perioperative SARS-CoV-2 infection, after careful cardiological evaluation.
  [Median score: 9 (*IQR* 7–9); agreement: 8/9 (88.8%)].
10. We emphasize the importance of patient basic assessment and evaluation in the increased risk, rather than just considering the timing and the 7-week timeframe. We support a multidisciplinary decision-making process.
  [Median score: 9 (*IQR* 7–9); agreement: 8/9 (88.8%)].
11. Parents/legal guardians of the pediatric patient awaiting surgery should collaborate to modify the risk factors of contagion, through compliance with prevention measures.
  [Median score: 9 (*IQR*: 7–9); agreement: 9/9 (100%)].

## Discussion

The current viral circulation characterized by Omicron variants and subvariants with very high contagion rate, combined with the widespread use of diagnostic tests and the high level of vaccination coverage, results in the probability that many patients with surgical conditions would enter the preoperative or perioperative period with a SARS-CoV-2 infection with forms of low clinical impact. These are patients who can be completely asymptomatic, paucisymptomatic, or can host the virus in the upper airways at an early stage, in which diagnostic techniques do not yet allow its detection [12]. In this context, the severity of COVID-19 infection with the Omicron variant appears to be milder than the previous variants [12, 46] as well as the hospitalization rate, the length of stay, and the mortality [11, 12, 47, 48].

These data suggest the hypothesis that the absolute risk of harm (morbidity or mortality), for the patient with recent SARS-CoV-2 infection to undergo surgery, could be lower than previous variants, but there are currently no robust evidence to confirm or dismiss this hypothesis. Therefore, on one hand, a previous SARS-CoV-2 infection with Omicron variant without symptoms or with mild symptoms could not be considered as a preoperative risk factor; on the other hand, it cannot be ignored that, with the previous variants, the SARS-CoV-2 infection, even asymptomatic, increased the risk of mortality approximately threefold during the 6 weeks following infection.

The indications that emerge from data related to the original virus and the first variants suggest that the interval between the diagnosis of SARS-CoV-2 infection and surgery should be at least of 7 weeks. The appearance of the Omicron variants, which generate less severe clinical pictures, combined with the significant increase in the number of immune subjects, due to the completion of the vaccination cycle, suggests the revision of the infection-surgery intervals, considering the possible negative consequences related to the postponement of the surgery. This group does not include subjects affected by SARS-CoV-2 with medium-severe symptoms, who required hospitalization or intensive care. For these subjects, it is mandatory to perform a careful evaluation of general clinical condition and organ functions, specially respiratory and cardiovascular [8]. It is therefore necessary to assess the risk of early surgery, or the possibility of delaying it, in light of the evolution of the pathology for which the surgery is scheduled. If this risk is lower than the clinical risk associated with the postponement of surgery, this delay would not be appropriate.

First, it is necessary to know the basic risk calculated using a validated risk assessment tool, such as the Surgical Outcome Risk Tool v2 (SORT-2) or the American College of Surgeon NSQIP Score [49]. The overall risk may be modified by a surgery performed within 7 weeks of the diagnosis of SARS CoV-2 infection, in light of the possible factors that modify the risk itself, such as patient-related factors (age, comorbidities, and functional status); SARS-CoV-2 infection (timing; variant, initial severity of infection, presence of symptoms); and, finally, factors related to surgery (clinical priority, risk of disease progression, complexity related to surgery itself) (E-Table 2 in the Additional file 1). Generally, patient categories with the greatest risk are oncology and all those for which there is significant clinical instability. The analysis of these risk factors is the basis of the decision-making process shared between the multidisciplinary team and the patient. The documentation should report the risks and benefits of the timing of the proposed surgery and the decision-making process.

Patients should be informed that the decision to proceed with surgery within 7 weeks will not be based on evidence but will be purely pragmatic, in the absence of reliable data.

The increased risk associated with surgical procedures after SARS CoV-2 infection is not reduced for up to 7 weeks; the risk posed by a referral is limited (for example, the increase in risk at 6 weeks after infection is similar to that at 3 weeks). Therefore, the decision-making process is dichotomic: to postpone for a period of 7 weeks or not to postpone. A multidisciplinary and personalized evaluation is therefore recommended. Elective surgery should be avoided during the period when a patient is likely to be most infectious (10 days), e.g., for patients who test positive for SARS-CoV-2 during preoperative screening (incidental SARS-CoV-2). Patients who are infectious represent a risk for healthcare professionals, for other patients, and for the whole pathway of care. Furthermore, incidental SARS-CoV-2 may be pre-symptomatic and may be associated with an increased risk of postoperative morbidity and mortality compared to patients undergoing elective surgery.

To our knowledge, there is no specific data on postoperative mortality and morbidity of children with previous SARS-CoV-2 infection, undergoing elective surgery. Considering the onset times of COVID-related pathologies in asymptomatic children with a history of previous infection, and while waiting for future evidence, it seems reasonable to support the same timing and the measures suggested for the adult patient. It is also reasonable to test for SARS-CoV-2 in the 48 h preceding the operation and re-evaluating the same day the possible appearance of symptoms. It is considered appropriate to postpone elective surgery for 2 weeks after vaccination for SARS-CoV-2, in consideration of the possibility to develop adverse effects (usually occurring after 1 week) with potential misinterpretation of the side effects of the vaccine and/or postoperative complications [50].

### Risk assessments should take place at the time of surgery planning

Patients must also be informed that the positivity to a preoperative SARS-CoV-2 test may trigger a review of the risks associated with the decision to proceed with the surgery anyway. A clear communication of the risk itself can be a valid support in the decision-making process, in relation to the correct timing possible (E-Table 2 in the Additional file 1). Some hospitals have adopted a 20-day waiting policy for positive but asymptomatic patients or 20 days after complete disappearance of symptoms [51]. The American Society of Anesthesiology (ASA) jointly with the Anesthesia Patient Safety Foundation formulated a joint statement in December 2020 which suggests 4 weeks of interval between infection and surgery for asymptomatic subjects, 6 weeks for symptomatic and 8–10 weeks for patients who required hospitalization or for diabetic patients, and finally, 12 weeks for those who have been hospitalized in the intensive care unit [52]. Even the scientific societies, both anesthetic and surgical dealing with outpatient surgery, have released documents in support of the criteria for selecting patients to be admitted to surgery after SARS-CoV-2 infection. The SAMBA statement of February 2021 agrees with the timing and methods of enrolling patients to undergo surgery, which have been defined for surgery in the ordinary hospitalization regime. It also underlines the importance of “prehabilitation” for these patients and above all the need for multidisciplinary teams, especially for fragile patients, if they can safely return home [53]. From a surgical point of view, the Spanish Association of Outpatient Surgery underlines the importance of identifying which patients really need surgery and which pathologies need to be managed surgically and which their level of priority. This model has been applied by many scientific societies, even though, by definition, the risk of interventions performed in outpatient surgery is moderately low [54].

For the correct assessment of the anesthetic and surgical risk, it is important to consider that in symptomatic or asymptomatic children with previous SARS-CoV-2 infection, alterations in cardiovascular function (i.e., reduction of left ventricular function, arrhythmias, coronary anomalies) may persist even after the resolution of the infection. Indeed, after the infection, symptoms attributable to MIS-C may also appear vague (conjunctivitis, musculoskeletal pain, easy fatigue) [21, 22]. Furthermore, there is also evidence of myocarditis following vaccination [55]. It is important, considering the scarce availability of reliable data, to perform a cardiological evaluation in all children with a history of previous SARS-CoV-2 infection. Since vaccine-induced heart disease is probably linked to autoimmune phenomena [56], cardiological evaluation is suggested. In some symptomatic cases, it has been reported that the symptoms may be present for a long time after the resolution of the infection, even for 15 weeks (long COVID) [57].

### Communicating the risk when considering surgery within 7 weeks of SARS-CoV-2 infection

The COVID-19 pandemic have had a major impact on the number of surgical procedures worldwide [58]. Under these conditions, it is expected that a significant number of patients candidates for elective surgery present the effects of a previous SARS-CoV-2 infection. Individuals undergoing surgery after recovering from COVID-19 have been shown to be at increased risk of complications and mortality. However, the longer the time between the diagnosis of SARS-coV-2 infection and surgery, the greater the progressive reduction in surgery-related complications. When this is performed after 7 weeks of diagnosis, the mortality is comparable to that of patients who have never contracted the infection (2.0% and 1.4%, respectively) [33]. Based on these data, the Royal College of Surgeons and the Royal College of Anesthetists recommend not to operate patients who have had asymptomatic Sars-CoV-2 infection or have overcome symptoms before 7 weeks [1]. For patients who have been hospitalized or with persistent symptoms related to SARS-CoV-2 infection, due to the significant risk of mortality affecting them, the surgery should be postponed beyond the 7-week limit. Elective surgery should be avoided during the period in which the patients are infectious since they represent a possible source of transmission of the virus to staff members and other patients. The necessary precautions should also be taken to prevent patients, especially those at high risk, from contracting the infection in the perioperative period. The size of this phenomenon, the positivity for SARS-CoV-2 found perioperatively, was defined in a systematic analysis conducted between March and December 2020 in the USA; the incidence stood at 5 positivity per 1000 procedures [44]. Female patients, those undergoing neurosurgery and those characterized by greater comorbidity, proved to have the highest probability of testing positive for SARS-CoV-2 in the immediate postoperative period [44]. An increased incidence of both major complications and lengthening of hospital stay was also found in this population [44].

In patient recovered from mild COVID-19, a candidate for elective surgery for a disease whose treatment is likely to be postponed without particular risks, the indication to postpone the intervention beyond the expected 7 weeks from the recovery from COVID-19 seems to have solid evidence. The situation regarding individuals who are candidates for surgery for whom the complexity of the disease has required hospitalization or who continue to present persistent symptoms is more complex. In this case, where the patient’s condition or the pathology that requires treatment does not allow postponing the surgical treatment beyond the limits that have proved to be of greater safety, it becomes necessary to proceed together with the patient to a careful evaluation of all those aspects that can contribute to the correct stratification of the risk related to that procedure.

The algorithm proposed by the panel allows a step-by-step definition of a cumulative risk, assessed on the basis of a score assigned in consideration of the age, the ASA status, and the type of surgery in addition to the weight to be assigned to the COVID-19 history of each patient. Aiming to comprehensive prognostic evaluation, some of the acute phase markers such as D-dimer, ferritin, and PCR have been evaluated. In some patients, they remain elevated, as a possible expression of persistent inflammatory state, up to 6–8 weeks after recovery from the disease [54]. Similarly, elevated values of NT-ProBNP and troponin with high sensitivity HSTn could be an expression of pulmonary and cardiac alterations that in a population of COVID-19 patients have been shown to be maintained beyond the viral phase [58, 59]. Further investigations are considered necessary to define to what extent these alterations affect recovery from COVID-19 [54, 58]. The identification of parameters that have prognostic importance should contribute to the better definition of predictive models able to quantify the perioperative risk more effectively [59] (Table 1).

**Table 1:** Preoperative assessment protocol for patients with previous COVID-19 based on the type of surgery and the severity index of the disease [51]

Step/test	Minor surgery		Major surgery	
	Asymptomatic	Symptomatic	Asymptomatic	Symptomatic
Chest X-ray	No, if SpO2 is normal	No, if SpO2 is normal	Yes	Yes
ECG	Yes	Yes	Yes	Yes
Echocardiography	No, when clinical condition is normal	No, when clinical condition is normal	No, if the results of cardiological tests are normal	Determined by H&P
Complete metabolic order	Yes	Yes	Yes	Yes
Complete blood count with formula	Yes	Yes	Yes	Yes
PTT	No	Based on clinical severity	Yes	Yes
D-dimer	No	Yes	Yes	Yes
Fibrinogen	No	Based on clinical severity	Yes	Yes
NT-proBNP	No	Yes	Yes	Yes
LDH, ferritin, prealbumin	No	Based on clinical severity	No	Based on clinical severity

*ECG*, electrocardiogram; *LDH*: lactate dehydrogenase; *SpO2*, peripheral oxygen saturation; *PTT*, partial thromboplastin time

### Isolation

The months of isolation at home, necessary to control the spread of the pandemic, have had a significant effect on millions of people with cardiovascular disease, diabetes mellitus, and cancer. The WHO stated that the habit of sedentary lifestyle imposed for 3 months, on individuals over the age of 70 years or carriers of comorbidities, could lead to an increase in mortality [36, 60]. Data confirmed a tendency to postpone medical consultations and to avoid access to the emergency department both for fear of contagion and to avoid overloading the health system. Isolation also influenced eating habits and physical activity and also led to an increase in anxiety states and mood alterations, which required an increased need for supportive drug therapies [61]. It has been reported that preoperative isolation for more than 3 days may be associated with an increased risk of postoperative pulmonary complications [62]. Patients should be advised to increase physical activity, in addition to preoperative exercise, and also include nutritional optimization and smoking cessation during isolation and throughout the preoperative period [62]. It will therefore be very likely to have to stratify the perioperative risk of populations of patients returning from the pandemic, with a history of contagion behind them, who need access to hospital to undergo surgery. These patients, especially the older ones, may also present comorbid states neglected due to the pandemic, recent appearance of anxious and/or depressive state, and a possible generic prolongation of the state of COVID-19 (long COVID). These patients, depending on the conditions and pathologies, will need to be faced with either the expected 7-week post-COVID-19 wait, able to reduce the risks of related complications, or the overall risk of an early intervention. It has been demonstrated that waiting for surgery may lead to worsening of anxiety and depression and may also favor their possible new onset [36].

To our knowledge, the literature does not provide evidence about the association of perioperative risk with the possible isolation of children in the days prior to surgery; therefore, it is not recommended to prescribe school leave.

### Anesthesia technique

In patients who contracted COVID-19, currently, there are no data supporting outcome differences between patients undergoing monitored anesthesia care (MAC), rather than locoregional anesthesia (ALR) or general anesthesia (AG) [63]. In a study conducted on 1128 patients undergoing noncardiac surgery, no differences were found in terms of mortality and incidence of respiratory complications at 30 days according to the type of anesthesia [64]. However, more recent evidence indicates the use of ALR versus AG techniques may be associated with a reduced risk of postoperative pulmonary complications and estimated mortality between 50 and 150% [33]. The potential advantages of ALR over AG were reported in a review that evaluated the management of obstetric anesthesia during the COVID-19 pandemic. Despite the limited amount of data available on COVID-19 infection, the authors confirmed that neuraxial analgesia for labor remains a cornerstone of obstetric care by limiting the exacerbation of respiratory symptoms associated with labor pain and the need for AG in case of cesarean section [64]. Particular attention should be paid to patients with previous neurological symptoms related to COVID-19 infection such as headache, disturbances of consciousness, paraesthesia, and other pathological signs that could potentially interfere with the follow-up of ALR [65]. The opioid-sparing effect of ALR can also be used in the preoperative management of a fragile patient [66]. In a case series, ALR was performed on 170 patients (97%) of the orthopedic patient population and on 43 (30%) of the remaining surgical patients. None of them showed complications related to the ALR technique [67]. Despite these potential advantages, the role of ALR techniques in the pandemic era needs to be carefully considered. In choosing the appropriate anesthesia technique, operators should also take into account the safety of ALR, related to the severity of the clinical presentation of SARS-CoV-2, the advantages in terms of drug-drug interactions, the side effects of experimental therapies for SARS-CoV-2, and the safety of the ALR regarding the clotting disorders typically exhibited by the COVID-19 patient. Considering these data, in patients with recent or actual SARS-CoV-2 infection in the perioperative phase, where appropriate characteristics exist, it may be considered to avoid AG, in favor of local or regional anesthesia techniques.

Regarding pediatric population, the anesthetic technique must always represent the best choice for the patient, in consideration of his clinical condition and the planned intervention. Since there is no evidence that the anesthetic technique impacts the onset of COVID-19-related postoperative complications, it is advisable to plan a strategy that minimizes the possibility of spreading any contagion escaped from routine perioperative checks. It should be considered that, in general, even in the case of locoregional anesthesia, the child requires sedation. In literature, it is possible to find suggestions about the routine application of pre-anesthesia in order to avoid crying and coughing with the potential for greater aerosol diffusion in the hospital environment [68, 69].

## Conclusions

The need to proceed to elective surgery must be balanced with the guarantee, for the patient, of being operated under safe conditions. There is currently a lack of data that provide specific information on perioperative risk. The decision to proceed to elective surgery after SARS-CoV-2 infection needs a weighted risk assessment, although in the indication to avoid surgery within 7 weeks of infection or in the event of symptoms it is necessary to evaluate the clinical priority and the risk of disease progression. Where it is possible to postpone the surgery, it is recommended to wait 7 weeks. Alternatively, if the surgical indication occurs within 7 weeks of infection or in the presence of symptoms, a multidisciplinary discussion should be initiated.

The risk assessment should consider patient’s specific factors and the status of the infection by providing an estimate of the overall risk. Rather than exclusively emphasizing the issue of surgical timing, it is important to highlight the need to link together timing, risk assessment, and shared decision-making. There is no data on the impact that a previous asymptomatic infection could have in the perioperative part of children undergoing elective surgery. In order to ensure safe healthcare, it is advisable to proceed as it was done in the pre-pandemic era, such as to undertake a careful interdisciplinary assessment of the perioperative risk and, when possible, postpone the surgery by 7 weeks from diagnosis of infection [1, 69].

In the pediatric field, dedicated courses are of great importance and should always include the possibility of guaranteeing the presence of the parent 24 h a day.

### Attachment 1 — flyer

#### Adult patient information: new timing instructions for elective surgery after SARS-CoV-2 infection

Your surgery may be postponed due to your SARS-CoV2 positivity. A new document analyzes the evidence and proposes some advice.

#### Why should my surgery be postponed?

Several studies conducted on populations of patients with COVID-19 infection, contracted immediately before or after undergoing surgery, have shown a higher incidence of complications as well as an increased risk of mortality. The risk of possible respiratory problems and changes in the clotting system is considered three or four times higher than normal for a period of 7 weeks after COVID-19 infection. This risk is also increased in patients who have contracted an asymptomatic infection.

#### How will I share with the doctors whether to continue or postpone the planned surgery?

You will be involved in a decision-making process. This means that an experienced doctor will speak out clearly about your case showing the risks, benefits, and possible alternatives. He will explain to you what could happen if you choose not to follow the recommended way (Table 2).

**Table 2:** What are the moments to remember?

Key points for adult patient	
Ten days before your surgery date	If you test positive, or have symptoms of COVID-19 infection, you should not proceed with elective surgery because you could infect other people and be exposed to higher risk of complications at the same time
Between 10 days and 7 weeks before your surgery date	•To reduce the personal risk, remember the social distancing, wear a mask, keep the windows open, or make sure you meet others outdoors •If you test positive, you should notify your healthcare team •It may be necessary to postpone the planned surgery •Doctors will evaluate your case to determine if it is more risky to postpone or proceed to surgery
Anytime before surgery	•Please try to keep yourself in good health and ready to face the surgery •Complete the vaccination course, including the third booster dose. Vaccination reduces both the chance of developing COVID-19 and the chances of transmitting the infection, and it also reduces the severity of the disease •Regular exercise, proper nutrition, and stopping smoking are measures that can reduce complications related to surgery by 30 to 80%. More information is available at the following address: www.cpoc.org.uk/patients

(E-Table 3 in the Additional file 1: Steps in the risk assessment process of adult patient)

### Attachment 2 — flyer

#### Pediatric patient information

##### Information for patient’s parents/legal guardians: new timing instructions for elective surgery following SARS-CoV-2 virus infection

Your child's surgery could be postponed in the event of positive COVID-19 test results. This document, based on scientific evidence, offers some advice.

#### Why should my surgery be postponed/delayed?

Studies conducted on populations of adult patients who contracted the virus immediately before or after undergoing surgery have shown that there is a higher incidence of complications, as well as an increased risk of mortality. In the adult patient, the risk related to possible respiratory problems and to those related to alterations of the coagulation system is considered at least three or four times higher than normal for a period that includes 7 weeks after COVID-19. This risk is also increased in patients who have contracted an asymptomatic infection.

For children, no reliable data is available, and although moderate and severe forms of COVID-19 are rare, it cannot be excluded that, over time, the infection results in a syndrome called MIS-C which causes alterations in heart function and other organs. This syndrome can be dangerous and increases the risk of complications from surgery and anesthesia (Table 3).

**Table 3:** What are the key points to remember?

	**Key points for pediatric patient**
Ten days before your surgery date	If your child test is positive, or he/she has symptoms of COVID-19 infection, it is advised to not proceed to elective surgery because he/she could infect other people and be exposed to higher risk of complications at the same time
Between 10 days and 7 weeks before your surgery date	•To reduce your child’s risk to contract COVID-19 infection when meeting other people, remember social distancing, wear a face mask, keep windows open, or make sure he/she meet others outdoors •If your child tests positive, you should notify their healthcare team •Scheduled surgery may need to be postponed •Doctors will evaluate the case to determine if it is more risky to postpone or proceed with the surgery
Anytime before surgery	•Please try to keep your child in good health and ready to face the surgery •If possible, make sure you have completed the vaccination course for the child, including the second dose •Vaccination reduces both the chance of developing COVID-19 and the chances of transmitting the infection, and it also reduces the severity of the disease

(E-Table 4 in the Additional file 1: Pediatric patient risk assessment process)

## Supplementary Information

**Additional file 1:** **E-Table 1.** Search strategy. **E-Table 2.** Communicate the risk when considering surgery within 7 weeks of SARS-COV 2 infection. **E-Table 3.** Steps in the risk assessment process of adult patient. **E-Table 4.** Pediatric patient risk assessment process. **Figure 1.** PRISMA flow 2020.
