# Supplementary material for: Timing of surgery and elective perioperative management of patients with previous SARS-CoV-2 infection: a SIAARTI expert consensus statement
Source: J Anesth Analg Crit Care. 2022 Jun 22;2:29. doi: 10.1186/s44158-022-00058-3 (PMC9214464; doi:10.1186/s44158-022-00058-3)
Supplement: Supplementary file 1 — Additional file 1: E-Table 1. Search strategy. E-Table 2. Communicate the risk when considering surgery within 7 weeks of SARS-COV 2 infection. E-Table 3. Steps in the risk assessment process of adult patient. E-Table 4. Pediatric patient risk assessment process. Figure 1. PRISMA flow 2020. [file 44158_2022_58_MOESM1_ESM.docx]

| **E-Table 1. Search strategy.** |
| --- |
| *Database:* PuBMed. |
| COVID AND SURGERY  (“sars cov 2”[MeSH Terms] OR “sars cov 2”[All Fields] OR “covid”[All Fields] OR “covid 19”[MeSH Terms] OR “covid 19”[All Fields]) AND (“surgery”[MeSH Subheading] OR “surgery”[All Fields] OR “surgical procedures, operative”[MeSH Terms] OR (“surgical”[All Fields] AND “procedures”[All Fields] AND “operative”[All Fields]) OR “operative surgical procedures”[All Fields] OR “general surgery”[MeSH Terms] OR (“general”[All Fields] AND “surgery”[All Fields]) OR “general surgery”[All Fields] OR “surgery s”[All Fields] OR “surgerys”[All Fields] OR “surgeries”[All Fields]) |
| *Time restriction:* From November 2021 to date (Filters to be applied to the search). |
| *Exclusion:* Not in English, Conference proceedings. |
| *Inclusion:* Original articles (any designs), case series, case reports, narrative reviews, systematic reviews, meta-analysis, position papers, guidelines. |

| **E-Table 2. Communicate the risk when considering surgery within 7 weeks**  **of SARS-COV-2 infection:** | | | |
| --- | --- | --- | --- |
| **STEP 1** | Assess BASIC RISK and inform the patient about it. | | |
| Basic Risk is the most important factor determining patient outcome | | | |
| Basic Risk | **HIGH**  Surgical mortality risk > 1 % with validated instrument2. | **INTERMEDIATE**  Low risk of death  but risk of serious complications. | **LOW**  Low risk of mortality and complications. |
| **STEP 2** | If you decide to proceed with surgery within 7 weeks assess the factors that determine ADDITIONAL RISK and inform the patient of this (increased risk factors). | | |
| Risk is cumulative: each risk factor has a more significant impact on a patient with a high baseline risk than a patient with a low baseline risk3.   - Age > 75 years - ASA status: 3- 5 - Major Surgery - Presence of Covid 19 infection symptoms. - Previous Hospitalizations for Covid 19 infection. | | | |
| Additional  risk | **HIGH**  >1 risk factor | **INTERMEDIATE**  1 risk factor | **LOW**  No risk factors |

| **STEP 3** | Assess the risk of postponing surgery after 7 weeks of Sars-Cov-2 infection. | | |
| --- | --- | --- | --- |
| Healthcare team and patients should consider the basic risk and the additional risk of procedeing with surgery and compare it with the risk associated with postponing the intervention. | | | |
| **STEP 4** | Complete the agreed result. | | |
| Outcome | Proceed | Postpone | Undecided |
| **Note** | | | |
| 1. Example of factors related to the patient and surgery associated with HIGH, INTERMEDIATE and LOW basic risk: | | | |
|  | Gastrointestinal system and hepatobiliary surgery, head and neck surgery, heart-lungs and great vessels surgery, complex surgery of the knee. | Other types of surgery: breast surgery, non-complex surgery of the skeletal system and soft tissues and plastic surgery. | Eye surgery and all minor surgery. |
|  | Fragile patient, with reduced functional capacity to carry out usual daily activities life and to care for the person;  patient with ongoing pathology or comorbidity. | Patient with moderate disability and without frailty. | Patient without disease and in good health. |
| 2. Basic risk assessment with a validated tool, SORT 2 is a suitable option [(http://www](http://www/). sortsurgery.com) | | | |
| 1. 3. Data from previous variants of SARS-CoV-2 showed that having one of these risk factors and performing surgery within six weeks of SARS-CoV-2 infection put them at more than doubled risk of complications and death. This risk is significantly reduced after 7 weeks. There are no evidence if this is applicable to the Omicron variant is currently unknown.    - Increasing age increases the risk, especially over 70 years.    - ASA status between 3-4 increases the risk approximately four times compared to ASA 1-2 patient.    - The presence of symptoms greatly increases the risk. Patients who have been hospitalized or who have ongoing symptoms require an individualized risk assessment.    - Major surgery has a two times higher risk than minor surgery. | | | |
| 4. ASA Score:  ASA 1 – A normal healthy patient.  ASA 2 – A patient with mild systemic disease without substantive functional limitations  (e.g current smoker).  ASA 3 – A patient with severe systemic disease with substantive functional limitations. One or more moderate to severe diseases.  ASA 4 – A patient with severe systemic disease that is a constant threat to life. ASA 5 – A moribund patient who is not expected to survive without the operation. | | | |
| 5. When it’s not possible to make a decision immediately, the patient should have more time to understand the risks and benefits of undergoing surgery within 7 weeks of Sars-CoV-2 infection. | | | |

| \| **E-Table 3. Steps in the risk assessment process of adult patient.** \| \| \| \| \| --- \| --- \| --- \| --- \| \| **1° step** \| **Establish the risk related to your clinical condition and the type of surgery planned according to the following scheme.** \|  \|  \| \| **Examples** \| **High risk** \| **Intermediate risk** \| **Low risk** \| \| **Types of surgery** \| Gastrointestinal system surgery, head and neck surgery, heart, lungs and great vessels surgery. \| Other types of surgery: breast surgery, non-complex surgery of the skeletal system and soft tissues and plastic surgery. \| Eye surgery and all minor surgery. \| \| Complex surgery of the knee, genitourinary system, skeletal system and soft tissues. \|  \|  \| \| **Patient** \| Fragile and severely disabled patients, whose clinical condition is linked to significantly compromised health conditions. \|  \|  \| | | | |
| --- | --- | --- | --- | --- | --- | --- | --- | --- | --- | --- | --- | --- | --- | --- | --- | --- | --- | --- | --- | --- | --- | --- | --- | --- | --- | --- |
| **2°step** | **Assessment of the additional risk represented by any problems related to the condition of recent COVID-19 disease.** | | |
| Each of the following risk factors adds weight to the calculated risk:   - - Age over seventy years;   - The concomitant presence of an alteration in the state of health;   - Major surgery;   - Failure to remit symptoms related to COVID-19 infection;   - COVID-19 disease requiring hospitalization. | | | |
| **3^°^ step** | **Risk assessment related to the postponement of the planned surgery.** | | |
| **4^°^ step** | **Only after full understanding of the overall risk will the result be shared.** | | |
|  | **High Risk** | **Intermediate Risk** | **Low Risk** |
| **Results:** | Surgery postponed for at least 7 weeks after COVID-19 infection or after positive test. | Green light for surgery. | Decision making without result. |

| **E-Table 4. Pediatric patient risk assessment process.** | | | |
| --- | --- | --- | --- |
| **1°step** | **Establish the risk related to the child clinical condition and the type of surgery planned according to the following scheme.** | | |
| Examples | High risk | Intermediate Risk | Low risk |
| Types of surgery: | -Gastrointestinal system surgery  -Head and neck surgery,  -Heart, lungs and great vessels surgery.  -Knee Surgery considered complex. -Genitourinary, skeletal systems and soft tissues surgeries. | Other types of surgery such as:  - non-complex surgery of the skeletal system and soft tissues;  - plastic surgery. | Eye surgery and all minor surgery. |
| Patients: | Fragile and disabled patients, in significantly compromised clinical conditions. | Patients with moderate disabilities, but without frailty. | Patients in good health. |
| **2° step** | **Calculate the additional risk posed by any problems related to the recent COVID-19 disease condition.** | | |
| Each of the following risk factors adds weight to the calculated risk:   - Age less than one year; - concomitant presence of any alteration in the state of health; - Major surgery; - Presence of symptoms related to COVID-19 infection; - Need for hospitalization during covid 19 infection; - MIS-C; - Worsening of heart function. | | | |
| **3° step** | Consider the risk associated with postponing surgery. | | |
| **4° step** | Proceed to share the result only after having fully understood the overall risk. | | |
| **Result:** | Surgery postponed at least 7 weeks after COVID-19 infection or test positivity. | Green light for surgery. | No decision. |


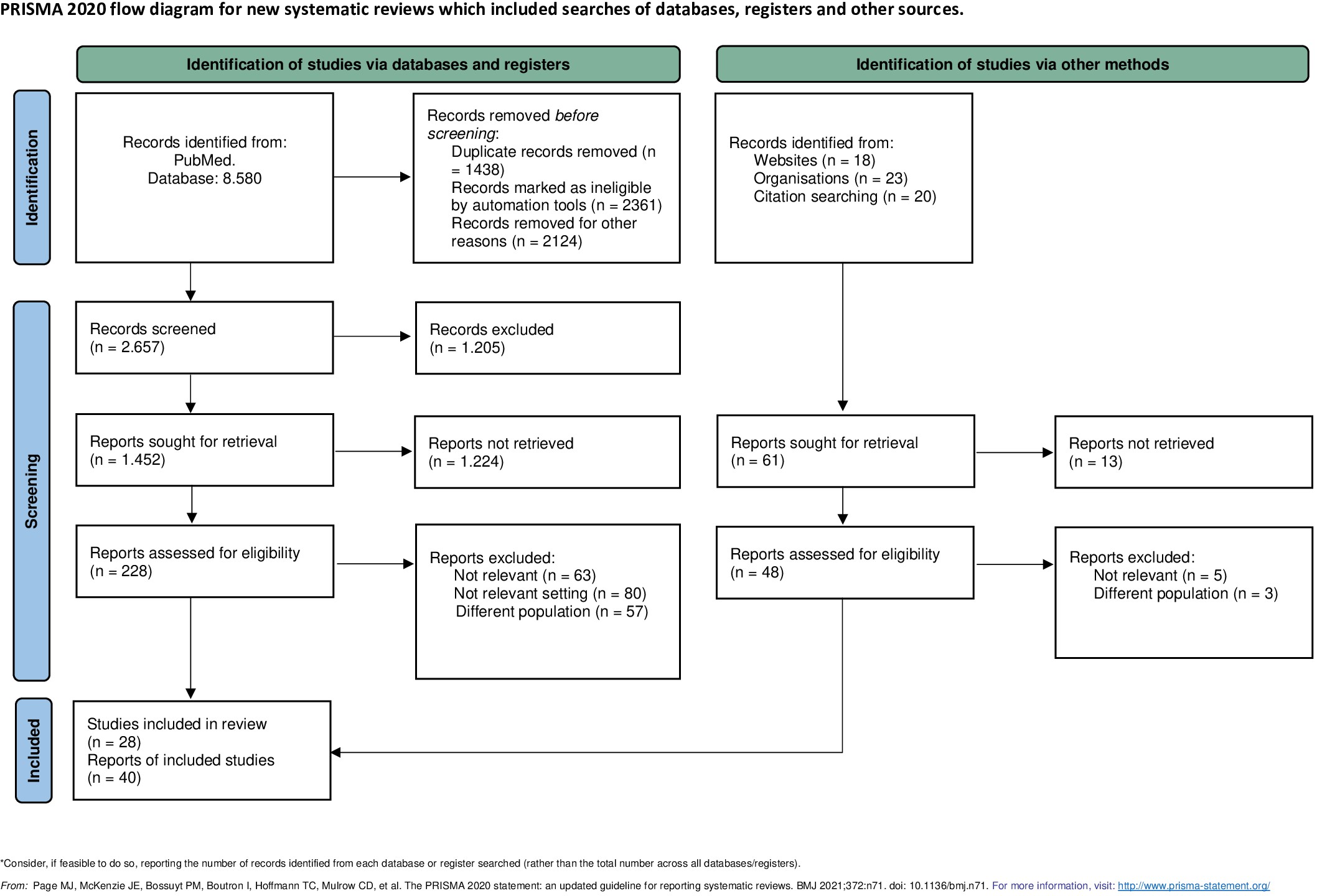
Figure 1.
